# Elevated interleukin-10, -22, -24, and -26 in serum samples of children with infectious mononucleosis

**DOI:** 10.5937/jomb0-54625

**Published:** 2025-07-04

**Authors:** Dalei Li, Kangwei Mao, Peiliang Luo, Ziqiang Zheng, Jiangyan Liu, Chenxi Zhang, Siyu Gu, Rui Zhang, Jun Sun, Juan Wang

**Affiliations:** 1 The Affiliated Lianyungang Hospital of Xuzhou Medical University (the First People's Hospital of Lianyungang, the First Affiliated Hospital of Kangda College of Nanjing Medical University, Lianyungang Clinical College of Nanjing Medical University), Department of Pediatrics, Lianyungang, Jiangsu, China; 2 The Affiliated Lianyungang Hospital of Xuzhou Medical University (the First People's Hospital of Lianyungang, the First Affiliated Hospital of Kangda College of Nanjing Medical University, Lianyungang Clinical College of Nanjing Medical University), Department of Clinical Pharmacy, Lianyungang, Jiangsu, China; 3 The Affiliated Lianyungang Hospital of Xuzhou Medical University (the First People's Hospital of Lianyungang, the First Affiliated Hospital of Kangda College of Nanjing Medical University, Lianyungang Clinical College of Nanjing Medical University), Department of Clinical Laboratory, Lianyungang, Jiangsu, China

**Keywords:** infectious mononucleosis, IL-10, IL-22, IL-24, IL-26, infektivna mononukleoza, IL-10, IL-22, IL-24, IL-26

## Abstract

**Background:**

Infectious mononucleosis (IM) is an infectious disease with different stages of onset, and the pathogenesis of IM remains unclear. This study aimed to investigate the levels of interleukin (IL)-10 family cytokines IL-10, -22, -24, and -26 in serum samples of patients with infectious mononucleosis (IM) and the potential diagnostic values.

**Methods:**

Thirty children with IM were enrolled in the study, and serum samples were collected; 30 healthy children served as the controls. Serum samples from the patients and healthy controls were collected, and IL-10, -22, -24, and -26 were determined by the ELISA method. An automatic biochemical analyser detected the serum alanine aminotransferase (ALT) and aspartate aminotransferase (AST). Moreover, potential diagnostic values of IL-10, -22, -24 and -26 have been analysed using ROC curve analysis, and the correlation between the levels of IL-10, -22, -24 and -26 in patients was analysed. Correlation between IL-10, -22, -24, and -26 and ALT and AST were analysed.

**Results:**

IL-10, -22, -24, and -26 levels increased in serum samples of patients with IM compared to healthy controls. ROC analysis showed that the AUC of IL-10, -22, -24, and -26 were 0.8500, 0.7078, 0.8056, and 0.9167, respectively, suggesting that IL-10, -22, -24, and -26 were good biomarkers. Moreover, IL-10, -22, -24 and -26 levels were positively correlated with the ALT and AST of the patients.

**Conclusions:**

IL-10, -22, -24, and -26 were upregulated in the serum of patients with IM, suggesting they may function as potential biomarkers.

## Abstract (sr)

**Uvod:**

Infektivna mononukleoza (IM) je zarazna bolest sa različitim fazama razvoja, a njena patogeneza još uvek nije u potpunosti razjašnjena. Ova studija imala je za cilj da ispita nivoe citokina iz porodice interleukina (IL)-10, -22, -24 i -26 u serumskim uzorcima pacijenata sa infektivnom mono nukleozom (IM) i njihovu potencijalnu dijagnostičku vrednost.

**Metode:**

U studiju je uključeno 30 dece sa IM i prikupljeni su serumski uzorci. Bilo je i 30 zdravih ispitanika kao kontrolna grupa. Nivoi IL-10, -22, -24 i -26 određeni su ELISA metodom, dok su vrednosti alanin aminotransferaze (ALT) i aspartat aminotransferaze (AST) merene pomoću automatskog biohemijskog analizatora. Pored toga, potencijalna dijagnostička vrednost IL-10, -22, -24 i -26 analizirana je pomoću ROC krive, a ispitana je i korelacija između nivoa IL-10, -22, -24 i -26 kod pacijenata. Tako|e je analizirana povezanost između IL-10, -22, -24 i -26 i nivoa ALT i AST.

## Introduction

Infectious mononucleosis (IM) caused by EB virus (EBV) infection is an acute systemic disease characterised by fever, pharyngitis, and lymphadenopathy. Although this disease is self-limiting and has a good prognosis, approximately 50% of the patients have liver damage, and severe cases can develop into hemophagocytic syndrome and other serious complications [1] [2]. Recently, the incidence of IM in children has been increasing. Because of children’s relatively immature immune systems, infection with EBV can easily cause T lymphocyte proliferation, leading to immune injuries. Moreover, the clinical manifestations of childhood IM are diverse and atypical, which can easily lead to missed diagnosis or misdiagnosis [3] [4]. Recently, the value of peripheral blood routine neutrophil to lymphocyte ratio (NLR), monocyte to lymphocyte ratio (MLR), and red blood cell distribution width (RDW) as inflammatory indicators in the diagnosis, treatment, and prognosis evaluation of autoimmune diseases, tumours, and respiratory system diseases has attracted widespread attention [5] [6]. However, further research is needed on the indicators used for early diagnosis of IM.

EBV is the primary pathogen causing IM. During the incubation period of infection, EBV mainly attaches to B lymphocytes. However, B lymphocytes do not express EBV nucleoprotein; thus, they do not have pathogenic ability [7]. However, if a minimal number of memory B lymphocytes differentiate into plasma cells, viral particles can be released, activating cytotoxic T lymphocytes (CTLs) and leading to a large release of inflammatory factors, including TNF-α, IL-1β, IL-6, IL-10, and other interleukins [8]. EBV changes from a latent state to a proliferative state, which can trigger a series of clinical symptoms. Xu et al. made discoveries by monitoring the expression changes of peripheral blood cytokines in children with IM. Changes in serum IL-17A, IL-22, Tim-3, and gal-9 levels are of great significance for evaluating the condition of children [9].

Additionally, 40 children with IM as the research group and 42 children with upper respiratory tract infections received treatment simultaneously with the control group. Serum levels of TNF-α, interferon-γ (IFN-γ), IL-6, and IL-10 IL-1 β in the research group were significantly higher than those in the control group [10]. Zu et al. [11] observed the role of CD_8_^+^CD_28_^-^ T regulatory cells in children with acute IM through flow cytometry and found that IL-6, IL-10, IFN-γ, ILT-3, and ILT-4 expressions in the serum of children with IM significantly increased. IL-10 is an important cytokine in the process of IM disease. Previous research has shown that IL-10 is a founding member of the cytokine IL-10 family. It is a multifunctional cytokine that limits excessive inflammatory responses, regulates innate and adaptive immune responses, promotes tissue repair, and plays an important role in maintaining tissue stability, especially during infection and inflammation. Currently, relevant studies are reporting the involvement of IL-10 in IM, but there is no report on the diagnostic value of the combined detection of IL-10, -22, -24, and -26 in children with IM.

Currently, we focused on the expression of the immunosuppressive factors IL-10, -22, -24, and -26. We hypothesised that IL-10, -22, -24 and -26 expressions were changed in patients with IM.

## Materials and methods

### Patients

The participants were 30 children with IM from the First People’s Hospital of Lianyungang between May 2021 and May 2023. Peripheral blood was collected from the patients before and after 14 days. Peripheral blood samples were collected from each patient at two time points: at the initial diagnosis, corresponding to the acute phase of the disease, and then collected again after a 14-day interval. The second sampling is to monitor the changes in cytokine levels in patients during the whole course of the disease. Considering the heterogeneity of individual rehabilitation models, this time range may include the late acute phase and early recovery period. The controls were 30 healthy individuals. This study was approved by the Ethics Committee of The First People’s Hospital of Lianyungang, and each participant signed an informed consent document.

Referring to the diagnostic principles of non-neoplastic EB infection-related diseases, all patients included in this study were newly diagnosed children with no previous history of EB infection. Patients should meet any three of the following clinical indicators and the fourth laboratory indicator: laboratory-confirmed cases; patients should meet any three of the following clinical indicators and any one of the three laboratory indicators: clinical indicators: 1) fever, 2) angina, 3) cervical lymphadenopathy, 4) splenomegaly, 5) liver enlargement, and 6) eyelid oedema. Laboratory indicators: 1) positive anti-EBV-VCA-IgM and anti-EBV-VCA-IgG, and negative anti-EBV-NA-IgG, 2) negative anti-EBV-VCA-IgM, but anti-EBV-VCA-IgG antibody was positive and belonged to the low-affinity antibody group, 3) titer of two serum samples with anti-EBV-VCA-IgG antibody increased by >4, and 4) peripheral blood heterotypic lymphocyte ratio 0.10 and/or lymphocytes 5.0×10^9^/L. This study excluded patients with other systemic diseases or non-collaborators.

### Specimen collection and preservation

Within 24 hours from admission and on days 7-10 of the disease course, 2 mL of peripheral venous blood was collected from children with IM and placed in a non-anticoagulant centrifuge tube. After natural coagulation at room temperature for 10 min, a high-speed freeze centrifuge was used to centrifuge at 3000 rpm for 10 min. 100 μL of upper serum was taken and frozen at -80°C for testing. The control group only needed to collect blood samples once within 24 hours from admission using the same method.

### ELISA

Cytokines were measured by enzyme-linked immunosorbent assay (ELISA) kits specifically for human interleukin -10 (IL-10), IL-22, IL-24, and IL-26, all from Beijing Baizhi Biotechnology Company. Each cytokine was analysed in strict accordance with the manufacturer’s plan. An ELX-800 multifunctional microplate reader (BioRad Company, USA) was used to measure each well’s optical density (OD) at the wavelength of 450 nm.

### ALT and AST levels

The subjects’ serum ALT and AST levels were detected using an automatic biochemical analyser (Shanghai Defu Biomedical Technology Co., Ltd., AS-690 model).

### Statistics

Data were analysed using SPSS software (SPSS Inc, Chicago, Illinois, USA). All data are presented as the mean ± standard deviation (x̄±SD). The Student’s t-test was used to compare the data of the two groups, and statistical significance was set at *P*<0.05. Receiver operating characteristic (ROC) curve analysis was used to identify optimal cut-off values of cytokines levels to identify with maximum sensitivity and specificity for detection of IM severity. Correlations were evaluated by calculating Pearson’s correlation coefficients.

## Results

### Elevated IL-10, -22, -24 and -26 in serum of patients with IM

**Figure 1:**
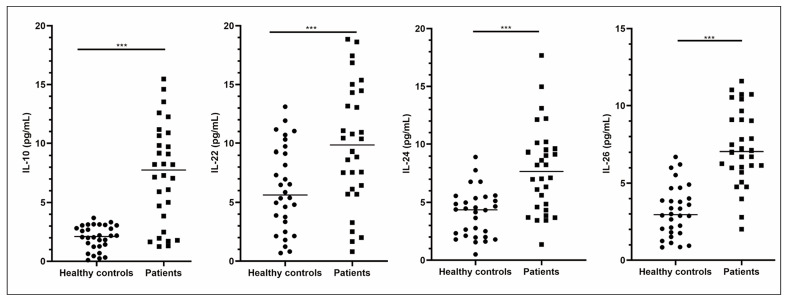
Up-regulation of IL-10, IL-22, IL-24 and IL-26 in serum of IM patients.<br>***P<0.001

We compared IL-10, IL-22, IL-24 and IL-26 concentrations in serum samples of patients with IM and healthy controls. IL-10, -22, -24, and -26 levels were markedly increased in the serum of patients with IM compared to healthy controls (Figure 1, P<0.001).

### IL-10, -22, -24 and -26 may distinguish patients with IM from healthy controls

**Figure 2:**
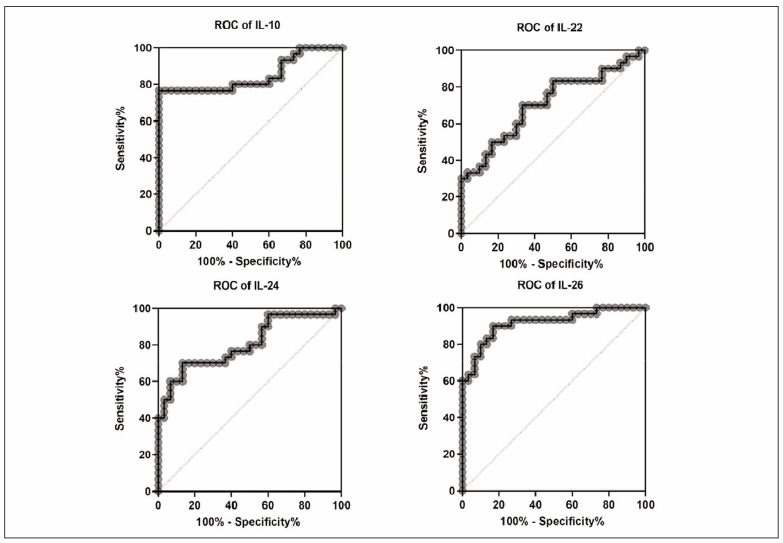
IL-10, IL-22, IL-24 and IL-26 may distinguish IM patients from healthy controls.

The potential diagnostic values of IL-10, -22, -24, and -26 for IM were evaluated. The ROC analysis showed that the AUC of IL-10, -22, -24, and -26 were 0.8500, 0.7078, 0.8056, and 0.9167, respectively (Figure 2 and Table 1). According to the Swets guidelines, the clinical accuracy of each examination parameter was classified as 0.5–0.7, which is a relatively low accuracy; 0.7–0.9, valuable accuracy for specific purposes 0.9, quite a high accuracy [12]. This indicates that IL-10, IL-22, IL-24, and IL-26 all have high accuracy, especially IL-26, which has a relatively high accuracy. Therefore, this suggests that IL-10, IL-22, IL-24, and IL-26 are good biomarkers for distinguishing IM patients from healthy controls.

**Table 1:** ROC curve analysis of cytokines to distinguish IM patients from healthy controls.

Area under the ROC<br>curve	IL-10	IL-22	IL-24	IL-26
Area	0.8500	0.7078	0.8056	0.9167
Std. Error	0.05405	0.06746	0.05698	0.03666
95% confidence<br>interval	0.7441 to 0.9559	0.5756 to 0.8400	0.6939 to 0.9172	0.8448 to 0.9885
P value	<0.0001	0.0057	<0.0001	<0.0001

### IL-10, -22, -24, and IL-26 were positively correlated with the ALT and AST of the patients with IM

**Figure 3:**
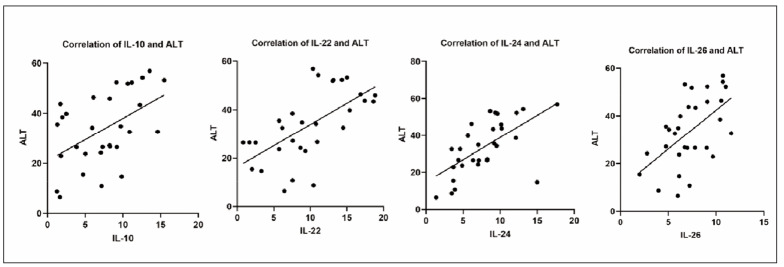
IL-10, IL-22, IL-24 and IL-26 were positively correlated with the ALT of the IM patients.

**Figure 4:**
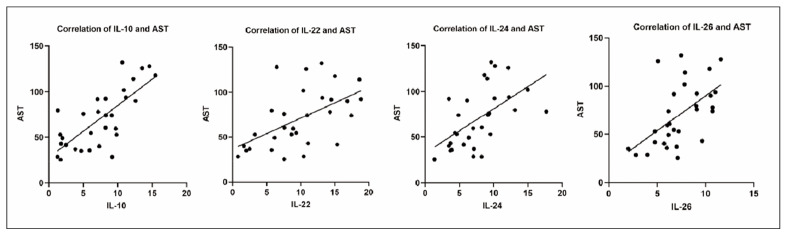
IL-10, IL-22, IL-24 and IL-26 were positively correlated with the AST of the IM patients.

Furthermore, the correlation between IL-10, -22, -24 and -26 levels and the biomarkers that could indicate the liver function of the patients was analysed. IL-10 (r=0.4979, p=0.0051), IL-22 (r=0.6253, p=0.0002), IL-24 (r=0.6255, p=0.0002), and IL-26 (r=0.5592, p=0.0013) levels were positively correlated with ALT of the patients (Figure 3). Furthermore, IL-10 (r=0.7451, p<0.001), IL-22 (r=0.5484, p=0.0017), IL-24 (r=0.5633, p=0.0012), and IL-26 (r=0.5563, p=0.0014) levels were positively correlated with the AST of the patients (Figure 4). Table 2 Table 3

**Table 2:** Correlation analysis of IL-10, IL-22, IL-24, IL-26 and ALT in IM patients.

Pearson r	IL-10	IL-22	IL-24	IL-26
r	0.4979	0.6253	0.6255	0.5592
95% confidence interval	0.1677 to 0.7276	0.3421 to 0.8044	0.3424 to 0.8045	0.2492 to 0.7653
R squared	0.2479	0.3910	0.3913	0.3127
P value				
P (two-tailed)	0.0051	0.0002	0.0002	0.0013
P value summary	**	***	***	**
Significant? (alpha=0.05)	Yes	Yes	Yes	Yes

**Table 3:** Correlation analysis of IL-10, IL-22, IL-24, IL-26 and AST in IM patients.

Pearson r	IL-10	IL-22	IL-24	IL-26
r	0.7451	0.5484	0.5633	0.5563
95% confidence interval	0.5261 to 0.8715	0.2345 to 0.7588	0.2548 to 0.7678	0.2451 to 0.7635
R squared	0.5552	0.3007	0.3173	0.3094
P value				
P (two-tailed)	<0.0001	0.0017	0.0012	0.0014
P value summary	****	**	***	**
Significant? (alpha=0.05)	Yes	Yes	Yes	Yes

## Discussion

Here, we explored the expression of the immunosuppressive factors IL-10, -22, -24, and -26 in patients with IM and observed that IL-10, -22, -24, and -26 were upregulated in their serum.

EB virus is a member of the lymphotropic virus genus in the herpesvirus family, and human herpesvirus is widely present in the population and commonly infected [13]. The positivity rate of EB virus antibodies in children aged 3–5 in China is >90%. EB virus-infected individuals and carriers can cause various diseases, such as IM, lymphoproliferative disorders, and nasopharyngeal carcinoma [14]. The typical clinical manifestations of IM are fever, swollen lymph nodes, and tonsillar pharyngitis, which can affect multiple body organs, causing organ function damage [2]. IM is a common infectious disease in children. With the continuous development of the disease, it can induce liver failure, seriously affecting the prognosis of the child [15]. Therefore, actively understanding the high-risk factors of IM and guiding clinical standardised treatment is significant for achieving a good prognosis in children.

Previous studies have shown that there are high levels of various cytokines in the serum of children with IM caused by EBV infection. Some authors have found that children with high EBV-DNA load in IM have more severe clinical symptoms, and IL-2 and -6 levels are positively correlated with EBV-DNA load, providing some reference for better differentiation of IM [4]. Subsequent studies have shown that multiple cytokines are involved in the disease process of IM. Zhang indicated that cytokines, Th1 cell marker IFN-γ, and Th2 cell marker IL-4, -6, and -10 were considerably lower in the patients with IM after antiviral treatment [16]. Li’s [17] study suggested that the IL-17 and -22 levels in the IM group were higher than those in the control group. IL-10 is recognised for its anti-inflammatory properties, while IL-22, IL-24 and IL-26 are related to T cell-mediated immune response and tissue repair. The level changes of these cytokines in autoimmune diseases indicate their potential as biomarkers or contributors to disease progress [18] [19]. At the same time, from a biological point of view, these interleukins play different but interrelated roles in regulating the activity of immune cells. For example, IL-10 can inhibit excessive immune activation as an autoimmune feature, while IL-22 and IL-24 promote tissue healing and barrier function [20] [21] [22]. Although little research has been done on IL-26, it has been proven to affect the balance of Th17/Treg cells, which is very important in autoimmune pathology [23]. Therefore, the imbalance or increase of these cytokines may affect the progress of IM by making the immune response promote or inhibit disease activities. Here, we found that IL-10, -22, -24, and -26 were markedly increased in the serum of patients with IM compared with healthy controls. The ROC analysis showed that IL-10, -22, -24, and -26 were good biomarkers for distinguishing patients with IM from healthy controls. Our research suggests that elevated serum IL-10, -22, -24, and -26 levels may be closely related to the occurrence and progression of IM, consistent with previous studies.

IM can affect multiple organs throughout the body, leading to functional damage to organs, such as the heart, liver, lungs, kidneys, pancreas, parotid gland, and brain, with liver function damage being the most common. Results of previous studies reported that the incidence of liver function impairment in children with IM is 80–90%. In contrast, Chinese survey data shows that approximately 50% of children with IM may experience liver function impairment [24] [25]. The primary manifestation of liver function impairment in children with IM is increased transaminases, including alanine aminotransferase, aspartate aminotransferase, and glutamine transferase. Therefore, understanding the high-risk factors associated with IM-induced liver dysfunction can provide a more comprehensive and in-depth understanding of its mechanisms of damage and guide clinical evaluation and treatment.

Additionally, we have discovered that IL-10, -22,-24, and -26 levels were positively correlated with the patients’ ALT and AST levels. Beata found that the serum level of IL-27 was significantly higher in children with mononucleosis than in healthy participants and positively correlated with ALT, AST, LDH activity, and WBC count [26]. These findings are consistent with our results.

Overall, we report for the first time that IL-10, -22, -24, and -26 are upregulated in the PBMCs of children with IM. The study results may provide new evidence for managing IM.

## Dodatak

### Funding

The funds supporting this study are from: Lianyungang First People’s Hospital Integrated Traditional Chinese and Western Medicine Research Project (Project Number: ZX8); Lianyungang Municipal Health Commission Research Project (Project Number: 2024011); Lianyungang »521 Project« Funded Project (Project Number: LYG065212024092); Lianyungang Municipal Health Commission Maternal and Child Health Research Project (Project Number: F202320); Lianyungang First People’s Hospital Young Talent Fund Project (Project Number: QN2317); Lianyungang Municipal Health Commission Research Project (Project Number: ZD202301).

### Conflict of interest statement

All the authors declare that they have no conflict of interest in this work.
